# Developing a Climate Change Risk Perception Model in the Philippines and Fiji: Posttraumatic Growth Plays Central Role

**DOI:** 10.3390/ijerph20021518

**Published:** 2023-01-13

**Authors:** David N. Sattler, James M. Graham, Albert Whippy, Richard Atienza, James Johnson

**Affiliations:** 1 Department of Psychology, Western Washington University, Bellingham, WA 98225-9172, USA; 2 Institute of Applied Sciences, University of the South Pacific, Suva, Fiji; 3 Department of American Ethnic Studies, University of Washington, Seattle, WA 98195-4380, USA; 4 The Weber Group, Suva, Fiji

**Keywords:** climate change, risk perception, posttraumatic stress, coping, resilience, natural disaster, hurricane, cyclone

## Abstract

Background: This two-study paper developed a climate change risk perception model that considers the role of posttraumatic growth (i.e., a reappraisal of life priorities and deeper appreciation of life), resource loss, posttraumatic stress, coping, and social support. Method: In Study 1, participants were 332 persons in the Philippines who experienced Super Typhoon Haiyan. In Study 2, participants were 709 persons in Fiji who experienced Cyclone Winston. Climate change can increase the size and destructive potential of cyclones and typhoons as a result of warming ocean temperatures, which provides fuel for these storms. Participants completed measures assessing resource loss, posttraumatic stress, coping, social support, posttraumatic growth, and climate change risk perception. Results: Structural equation modeling was used to develop a climate change risk perception model with data collected in the Philippines and to confirm the model with data collected in Fiji. The model showed that climate change risk perception was influenced by resource loss, posttraumatic stress, coping activation, and posttraumatic growth. The model developed in the Philippines was confirmed with data collected in Fiji. Conclusions: Posttraumatic growth played a central role in climate change risk perception. Public health educational efforts should focus on vividly showing how climate change threatens life priorities and that which gives life meaning and can result in loss, stress, and hardship. Disaster response organizations may also use this approach to promote preparedness for disaster threats.

## 1. Introduction

*“We are in the fight of our lives, and we are losing.”*

—Antonio Guterres, Secretary-General of the United Nations, November 2022

At the 2022 United Nations Climate Change Conference in Sharm El-Sheik, Egypt, the Secretary-General of the United Nations, Antonia Guterres, painted a bleak picture of Earth’s future unless immediate action is taken: “We are on a highway to climate hell with our foot still on the accelerator…. Greenhouse gas emissions keep growing, global temperatures keep rising, and our planet is fast approaching tipping points that will make climate chaos irreversible” [1]. Climate change is altering the climate system worldwide and is responsible, in part, for increasing the intensity of hurricanes (also referred to as cyclones or typhoons), heatwaves, and extended periods of drought in Australia, the Amazon, southern and western Africa, and the United States, and flooding in the United Kingdom, Bangladesh, and other South Asian countries [2,3,4]. These and other climate change-related disasters threaten lives and property and affect the physical well-being, mental health, and livelihoods of tens of millions of people worldwide. Climate change is considered to be “a true public health emergency” [5,6].

This paper developed a model of climate change risk perception with participants in the Philippines who experienced Super Typhoon Haiyan and attempted to confirm the model with participants in Fiji who experienced Cyclone Winston. This approach allowed us to examine whether the model developed in one country would replicate in another country and thus consider its generalizability. These storms were among the strongest in recorded history to make landfall on these island nations. There was significant discussion concerning the role of climate change in increasing the strength and magnitude of each storm, primarily as a result of increasing ocean temperatures [7,8]. Warming ocean waters fuel typhoons, cyclones, and hurricanes and can intensify their destructive potential: larger size, stronger wind speed, and higher storm surge [3,9]. Examining factors that influence climate change risk perception is essential (a) given that despite overwhelming scientific consensus, some portion of the public and politicians question the existence of climate change and the scientific consensus concerning the role of human activity in climate disruption, and (b) for developing approaches to reduce carbon footprints [10,11]. As such, our climate change risk perception measure assessed cognitions regarding how climate change can create storms with greater destructive potential and concern for their consequences on livelihoods [5,10].

Super Typhoon Haiyan created extensive devastation along coastal areas in the Philippines and was the second-deadliest storm in recorded history to strike the country, with 6293 persons who lost their lives and 28,689 persons injured. Property damage estimates were close to USD 1 billion, 1,140,332 homes were damaged or destroyed, and 890,895 families (or 4,095,280 million persons) were left homeless [12]. It destroyed significant portions of the ecosystem, including 33 million coconut trees and crops [13].

Fifteen months after Super Typhoon Haiyan, Cyclone Winston devastated communities on 167 of the 330 islands throughout Fiji. Cyclone Winston was one of the strongest cyclones ever recorded in the Southern Hemisphere and the strongest cyclone to have made landfall in Fiji. Just under half of the population of Fiji (40%) was impacted by the cyclone, and 43 persons lost their lives [14]. The cyclone damaged or destroyed 28,000 houses and leveled nearly all of the buildings in some communities and villages [15]. Property damage estimates exceeded USD 470 million, or close to 10% of Fiji’s gross domestic product [16]. The cyclone destroyed significant portions of the ecosystem, including crops and coconut trees.

### Developing a Climate Change Risk Perception Model

In the present study, we developed a psychological model of climate change risk perception in the Philippines with data collected after Super Typhoon Haiyan (Study 1). We attempted to confirm the model with data collected in Fiji after Cyclone Winston (Study 2). Conservation of resources stress theory (COR) guided our model development, as it provides a useful framework for examining the mental health impact of disaster exposure and considering how such experiences may be associated with climate change risk perception [17]. COR underscores the importance of an array of resources for physical and mental health and identifies four domains of resources: object resources (e.g., house, possessions), condition resources (e.g., employment), personal characteristic variables (e.g., self-esteem, optimism), and energy resources (e.g., time). According to COR, stress can result when resources are threatened, damaged, or destroyed. The inability to replace lost resources can exacerbate the stress response [17]. Notably, resource loss spirals can develop wherein the loss of resources in one domain results in a threat to or loss of resources in other domains. For example, people who experienced Super Typhoon Haiyan or Cyclone Winston experienced life threats during the storm, loss of income due to loss of employment, and inability to access social support networks due to infrastructure damage (e.g., loss of electricity, road closure, and inoperative telephone networks). Associated losses may adversely influence personal characteristic variables, such as decreased sense of self-efficacy and optimism. Resource loss spirals can create new stressors that overload remaining resources and negatively impact mental health; they are especially problematic when basic resources for survival—food, water, shelter, and clothing—are completely lost, and extreme damage to community infrastructure creates significant barriers and challenges to obtaining them.

Resource loss and life threats as a result of a disaster increase the possibility of developing posttraumatic stress symptoms, which include flashbacks, nightmares, anxiety, and uncontrollable thoughts about the event [18]. These responses may activate coping responses, including problem-focused coping and posttraumatic growth. Problem-focused coping involves attempts to change or manage the situation directly, and it can help survivors reestablish feelings of control and obtain and replenish resources. Problem-focused coping has been associated with posttraumatic growth among persons who experienced disasters [19,20].

Posttraumatic growth involves reappraisal and reassessment of one’s values and life priorities and reinterpreting the event by giving it new meaning [21]. Several studies have documented posttraumatic growth among disaster survivors, including the development of new coping skills, a heightened sense of self-efficacy, and gaining a new appreciation for others, especially during the first few months following a disaster when people often report making friends when receiving and providing assistance. These and other positive outcomes help counter negative experiences [20,21,22].

This two-study project is among the first to develop a model of climate change risk perception that considers the role of resource loss, psychological distress, coping and social support, and posttraumatic growth in the wake of catastrophic weather events whose strength was associated with climate change. Because posttraumatic growth involves considerable reflection of one’s values and life priorities, we are particularly interested in how this response may be associated with a heightened concern for or increased sensitivity to potential new threats following an experience with a catastrophic cyclone or typhoon. In so doing, our study extends research showing that personal experience with and knowledge of extreme weather events, especially in one’s local area, provides information about climate change threats [23,24,25]. 

Based on COR and prior research, we hypothesized that the experience of life threats and loss of resources as a result of the typhoon or cyclone would activate posttraumatic stress reactions, which would then activate coping responses (including social support), followed by posttraumatic growth reactions [10,17,25,26]. We predicted posttraumatic growth reactions would be directly associated with climate change risk perception. This hypothesis is based on the consideration that the process of reflecting on one’s life priorities associated with posttraumatic growth would increase sensitivity and concern regarding potential new threats.

## 2. Method (Study 1, Philippines)

### 2.1. Participants

The participants were 332 persons (102 men and 230 women) in coastal communities near Ibajay, Aklan, Philippines (age *M* = 42, *SD* = 20, range = 18–89 years). Most were married (65%), followed by the status of single (24%), widowed (7%), and separated/divorced (3%). Most were Catholic/Christian (94%), followed by other (3%), Iglesia ni Kristo (2%), and Buddhist (1%). About one-third (37%) had less than a secondary school education or completed secondary school (36%), and about one-quarter (27%) had some college or a college degree.

### 2.2. Assessment Instruments

To translate the assessment instruments from English to Tagalog, we used a version of the committee approach that we have successfully used in prior studies [10,22,26,27,28,29]. The fourth author translated the items, a consultant revised the translation, and a teacher and two medical nurses in the Philippines evaluated the instruments. A cover letter introduced the study, presented informed consent information, and indicated that responses were anonymous. The instruments were presented in the following order.

*Demographics, prior disaster experience, and life threat*. Nine items asked for demographic information. Three items asked about prior disaster exposure. An example is “How much damage has a natural disaster other than Cyclone Winston caused to your home.” Participants used a 5-point scale (0 = no damage to 5 = total home loss). For other items, participants checked their choices or wrote in a number to indicate their answers.

*Resource loss*. An 18-item scale assessed the loss of personal characteristics (e.g., sense of optimism, hope), object (e.g., food, home furnishings), condition (e.g., support from co-workers, companionship), and energy (e.g., time for adequate sleep, motivation to get things done) resources in the past four weeks [26,30]. Participants used a 5-point scale (0 = no loss to 4 = great loss) to indicate their answers. We summed items to create a resource loss scale total score, where higher values indicated a greater level of resource loss. The scale reliability was excellent, α = 0.92.

*Posttraumatic stress and somatic problems*. The 22-item Impact of Event Scale-Revised assessed symptoms associated with posttraumatic stress disorder experienced in the past seven days (α = 0.94) [31]. Examples of items include “I felt irritable and angry,” “I stayed away from reminders about the cyclone,” and “Reminders of the cyclone caused me to have physical reactions, such as sweating, trouble breathing, nausea, or a pounding heart.” Five items assessed somatic problems. Higher scale scores indicate greater levels of symptoms. Participants used a 5-point scale (0 = not at all to 4 = extremely) to indicate their answers on both scales.

*Social support*. Seven items from the Social Support Index assessed support [32]. Examples include “If I had an emergency, even people I do not know in this community would be willing to help” and “People here know they can get help from the community if they are in trouble.” Participants used a 5-point scale (1 = not at all to 5 = very much). Higher scores indicate a greater level of social support. Scale reliability was very good, α = 0.85.

*Coping*. Six items adapted from the Family Crisis Oriented Personal Scale assessed problem-focused coping [32]. Examples include “I face problems head on and try to get the solutions right away” and “I know I have the ability to solve major problems.” Participants used a 5-point scale (1 = not at all to 5 = very much). The scale reliability was good, α = 0.76.

*Posttraumatic growth*. Sixteen items from the Posttraumatic Growth Inventory assessed the degree of growth experienced by participants [33]. Examples include “Priorities about what is important in my life” and “Appreciating each day.” Participants used a 7-point scale (1 = great decrease, 4 = no change, and 7 = great increase). Higher scores indicate a greater degree of posttraumatic growth. The scale reliability was excellent, α = 0.93.

*Climate change risk perception*. Eight items written by the first author asked about perceived risks associated with climate change and the degree to which participants felt climate change contributed to the intensity of the typhoon. Examples include “I believe future typhoons will be stronger than normal because of climate change” and “I believe that global warming—the rise in the world’s temperatures—can create stronger typhoons that are more destructive to homes and property.” Participants used a 5-point scale (1 = not at all to 5 = very much) to indicate their answers. We summed the items to create a total score, with higher scores indicating stronger climate change risk perception. The scale reliability was very good, α = 0.82.

### 2.3. Procedure

We administered the survey five weeks after Super Typhoon Haiyan made landfall in three seaside villages near Ibajay, a small city of 45,279 people in the rural province of Aklan, Philippines. Each village and the surrounding area experienced significant destruction due to the typhoon.

The first author trained two nurses, a teacher, and three Red Cross volunteers in questionnaire administration. Local disaster officials informed the research team of the seaside villages that experienced extensive damage. Sampling involved soliciting participation from adults 18 years of age or older who resided in these locations. If no one was home, we returned one time. The sample target was 300 residents. Most completed the assessment instruments with no assistance (71%), with the remainder requiring some items to be read to them. The questionnaire took about 35 min to complete. Participation was voluntary, no inducements were offered, and the response rate was 97%; almost all people agreed to complete the survey. This study was approved by the Human Participants Research Committee at Western Washington University and followed the American Psychological Association’s ethical guidelines.

## 3. Results (Study 1, Philippines)

### 3.1. Model Development Overview

We developed and tested models using Amos (version 21) [34]. We used maximum likelihood to estimate all parameters. Because of discussion in the literature concerning which fit indices are most appropriate, we employed a variety of methods: the Chi-Square test, the Root-Mean-Square Error of Approximation (RMSEA), the Standardized Root Mean Residual (SRMR), and the Comparative Fit Index (CFI). The Chi-Square test examines the null hypothesis that the data did not fit the hypothesized model. Statistically significant Chi-Square values indicate poor model fit. Chi-Square is of particular use for comparing the relative fit of nested models. The RMSEA is a commonly used measure of fit that rewards parsimonious models. The RMSEA has a minimum value of 0, with lower numbers indicating a better fit. Commonly used cut-off points for RMSEA values use 0.01, 0.05, and 0.08 to indicate excellent, good, and poor fit, respectively [35]. The SRMR is a measure of absolute fit, with values less than 0.08 indicating a good fit [36]. As a measure of the incremental fit, we used the CFI, which generally has a maximum value of 1 (though values can sometimes be larger), with larger numbers indicating a better fit. A CFI of 0.95 or 0.96 greater is typically considered a good fit [36].

### 3.2. Model Development 

Three hundred and twenty-one individuals had no missing data and were used in the analyses. We randomly split our data in half. We used one sample of 160 people to assess the initial model fit and to conduct a specification search and the remaining sample of 161 individuals to confirm the fit of the final model. The means, standard deviations, and correlations between the measured variables for the two samples are shown in Table 1.

Using the initial sample of 160 individuals, we first tested the fit of a measurement model with two observed variables and three latent variables with two indicators each: resource loss, posttraumatic symptoms, and coping. We used personal characteristic/condition loss and object/energy loss as indicators for the latent resource loss variable. We used somatic and posttraumatic stress symptoms as indicators for the latent posttraumatic symptom variable. We used social support and problem-focused coping as indicators for the latent coping variable. We used a single measured variable for posttraumatic growth and climate change risk perception. We allowed the three latent variables and two single observed variables to correlate with one another. Table 2 shows the resulting fit indices for the initial measurement model, Model 1. While the SRMR indicated an acceptable fit, the X^2^, RMSEA, and CFI indicated a poor fit. The resulting modification indices indicated that the model fit could be improved by allowing the error terms for personal characteristic/condition loss (e2) and social support (e6) to correlate. The personal characteristic/condition loss variable includes items reflecting loss of companionship, loss of intimacy, and loss of support from co-workers. Such losses would also presumably reduce the amount of possible social support available to an individual. This is consistent with the idea that some stressors directly act to reduce available coping resources. When these error terms were allowed to correlate in Model 2, the resulting fit indices, shown in Table 2, indicated an excellent fit.

We re-specified the measurement model into a structural regression model, converting the correlations between the variables into direct regression paths and adding error terms to any endogenous variables. We created a chained-mediational model in which resource loss preceded posttraumatic symptoms, followed by coping, posttraumatic growth, and finally, climate change risk perception. As seen in Table 2, this structural regression model, Model 3, is equivalent to the previous measurement model.

**Figure 1:**
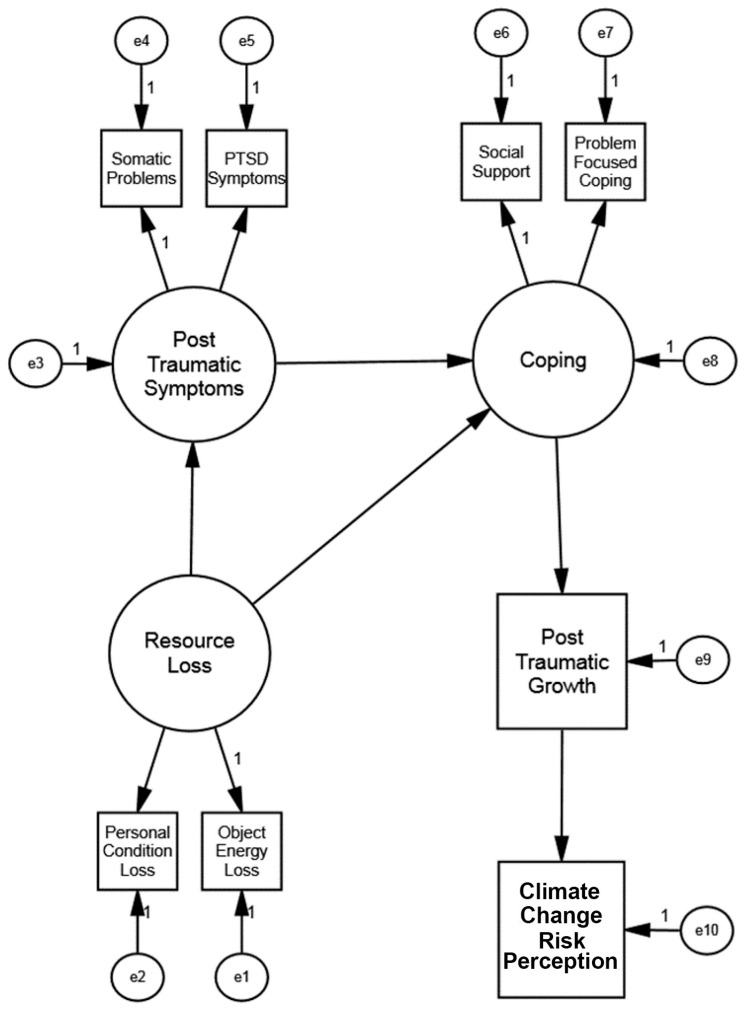
**(Study 1, Philippines)**. Initial Hypothesized Model: Influence of Resource Loss on Posttraumatic Symptoms, Coping, Posttraumatic Growth, and Climate Change Risk Perception.

Next, we performed a specification search. We examined the critical ratios for each of the regression paths and removed the regression path with the smallest critical ratio. We compared the fit of the resulting model to that of the full structural regression model to ensure there had not been a statistically significant change in the fit. We continued this process for subsequent regression paths until just before the fit of the model became statistically significantly worse than the full structural model. The paths removed (in order) were: Posttraumatic Symptoms => Posttraumatic Growth, Coping => Climate Change Risk Perception, Posttraumatic Symptoms => Climate Change Risk Perception, Resource Loss => Climate Change Risk Perception, and Resource Loss => Coping. The final model, Model 4, is shown in Figure 1, with the resultant fit indices in Table 2. Each of the resulting paths was statistically significant (*p* < 0.001). All fit indices of the final Model 4, shown in Table 2, were within the acceptable range. The fit of the final model was not statistically significantly different than the fit of the full structural regression model.

**Figure 2:**
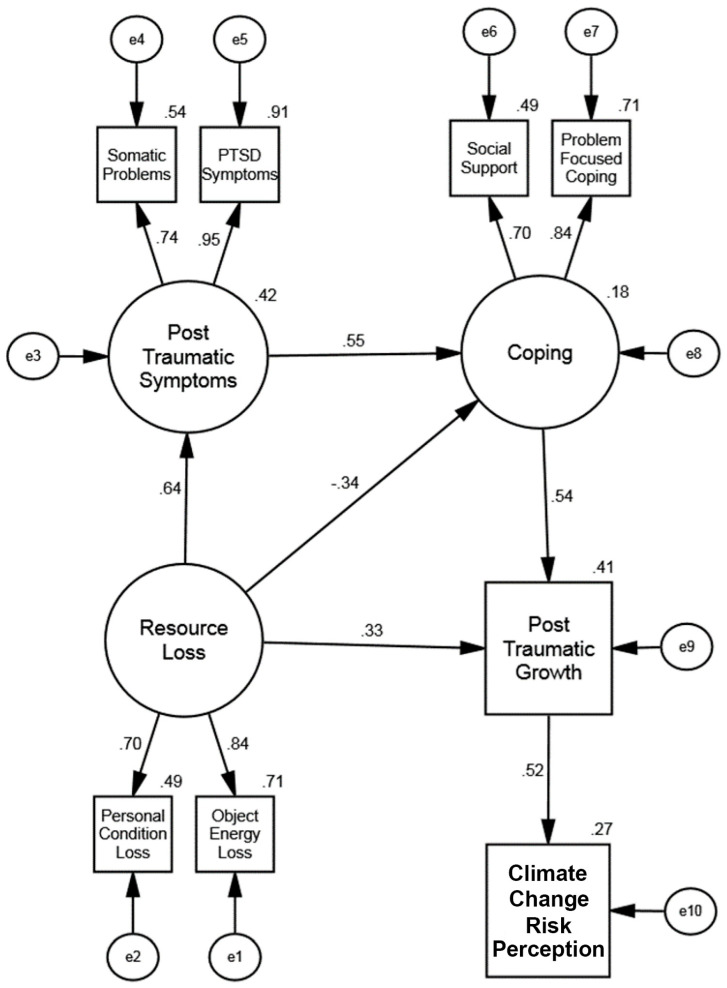
**(Study 1: Philippines)**. Final Structural Equation Model: Influence of Resource Loss on Posttraumatic Symptoms, Coping, Posttraumatic Growth, and Climate Change Risk Perception. The offset values on endogenous variables are R^2^ effect sizes. While not shown here, the error terms e2 and e6 were allowed to correlate (*r* = −0.34).

We used the data from the second sample of 161 individuals to confirm the fit of the final structural model, Model 4. The fit indices, shown in Table 2, indicate an excellent overall fit. Figure 2 shows the values for the path coefficients and the R^2^ for each endogenous variable. All structural paths were statistically significant (*p* < 0.001). Resource loss was positively associated with posttraumatic stress symptoms and posttraumatic growth. The results indicated that the more loss an individual experiences, the greater their potential for both posttraumatic symptoms and posttraumatic growth. The experience of posttraumatic symptoms was, in turn, associated with higher levels of coping. More coping was associated with greater posttraumatic growth. Resource loss was also directly associated with opportunities for posttraumatic growth, outside of the effect mediated by posttraumatic symptoms and coping. Finally, posttraumatic growth has a positive and direct relationship with climate change risk perception, with higher levels of posttraumatic growth relating to a higher degree of perceived risk due to climate change.

## 4. Brief Discussion (Study 1, Philippines)

Study 1 developed a model of climate change risk perception with persons in the Philippines who experienced Super Typhoon Haiyan. Importantly, the model documented variables contributing to and the importance of posttraumatic growth—reappraising and reassessing one’s values and life priorities and reinterpreting the event by giving it new meaning—for climate change risk perception. To confirm the model, we conducted Study 2 in Fiji, a country in the South Pacific, with participants who experienced Cyclone Winston, a disaster unrelated to Super Typhoon Haiyan, whose magnitude was associated with climate change.

## 5. Method (Study 2, Fiji)

### 5.1. Participants

The participants were 709 persons (337 men, 371 women, and seven unreported genders) in coastal communities in the Ra province of Fiji (age *M* = 41, *SD* = 16, range: 18–88 years). Most were married (65%), followed by the status of single (23%), widowed (8%), and separated/divorced (4%). Most were Catholic/Christian (84%), followed by Hindu (8%), religions not included on the list (6%), Muslim (2%), and Buddhist (0.3%). Nearly half (49%) had less than a secondary school education, about one-third had completed secondary school (34%), and about one-fifth (17%) had some college or a college degree.

The participants lived in 12 rural villages, and the population of each the village ranged from approximately 100–200 people. Each village and the surrounding area experienced significant destruction as a result of the cyclone.

### 5.2. Assessment Instruments

Participants in Fiji completed the same assessment instruments as those in the Philippines. The survey was in English, as English is an official language in Fiji and is taught in schools and regularly spoken throughout Viti Levu, the main island in Fiji.

### 5.3. Procedure

We administered the survey four weeks after Cyclone Winston made landfall. The first author trained 20 advanced university students in questionnaire administration. Local disaster officials informed the research team of the villages that experienced moderate-to-extensive damage, and we surveyed these villages. Sampling involved soliciting participation from adults 18 years of age or older. The response rate was 96%; almost all people agreed to complete the survey. Most completed the assessment instruments with no assistance (58%), with the remainder requiring some items to be read to them, and it took about 35 min. Participation was voluntary, and no inducements were offered. This study was approved by the Human Participants Research Committee at Western Washington University and the Institutional Review Board at the University of the South Pacific and followed the American Psychological Association’s ethical guidelines.

## 6. Results (Study 2, Fiji)

### Model Confirmation

The data collected in Fiji after Cyclone Winston (Study 2) was used to confirm the model developed with data collected in the Philippines after Super Typhoon Haiyan (Study 1). We randomly split the Fiji data in half and used one sample (*N* = 343) to assess the initial model’s fit and to conduct a specification search and the second sample (*N* = 343) to confirm the final model’s fit. Participants with missing data (*N* = 43) were not included in the analysis. Table 3 presents the means, standard deviations, and correlations between the measured variables for the two samples.

We first tested the fit of a measurement model with four observed variables and one latent coping variable with two indicators, each with the initial sample. We used a single measured variable for personal characteristics/conditions loss, posttraumatic stress symptoms, posttraumatic growth, and climate change risk perception. We used social support and problem-focused coping as indicators for the latent coping variable. We initially included object/energy loss as an indicator of latent loss, but preliminary psychometric analyses supported its removal. We allowed the latent variables and four single observed variables to correlate with one another. Table 4 shows the resulting fit indices for Model 1, the initial measurement model. While the SRMR and CFI suggested an acceptable level of fit, the X^2^ and RMSEA indicated a poor fit. The resulting modification indices indicated that the model fit could be improved by allowing personal characteristic/condition loss and the error term for social support (e2) to correlate. The personal characteristic/condition loss variable includes items reflecting loss of companionship, loss of intimacy, and loss of support from co-workers. Such losses would presumably also reduce the amount of possible social support available to an individual. This is consistent with the idea that some stressors directly act to reduce available coping resources [32]. When these error terms were allowed to correlate in Model 2, the resulting fit indices indicated an overall acceptable level fit (see Table 4). While the X^2^ was statistically significant, it was only just so, and the other fit indices were well within acceptable ranges. The resulting modification indices suggested that no further changes would substantially improve the model fit.

We re-specified the measurement model into a structural regression model, converting the correlations between the variables into direct regression paths and adding error terms to any endogenous variables. We ordered the variables in relation to COR and created a chained-mediational model in which personal characteristics/conditions loss preceded posttraumatic stress symptoms, followed by coping, posttraumatic growth, and climate change risk perception. Table 4 shows that this structural regression model, Model 3, is equivalent to the previous measurement model.

**Figure 3:**
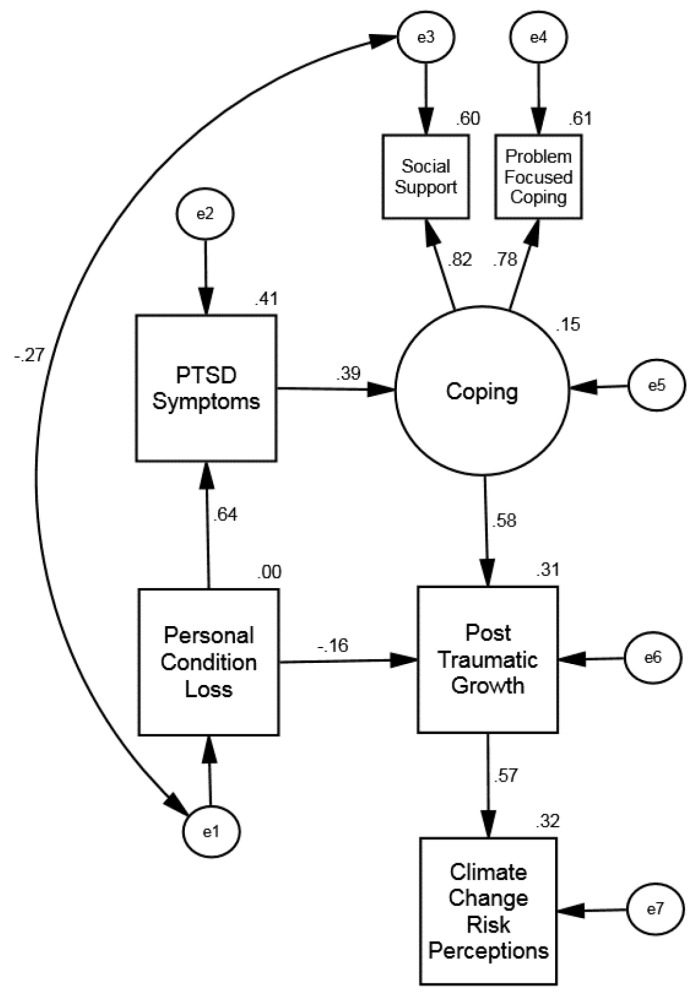
**(Study 2: Fiji):** Exploratory Sample Final Structural Equation Model: Influence of Resource Loss on Posttraumatic Symptoms, Coping, Posttraumatic Growth, and Climate Change Risk Perception. The offset values on endogenous variables are R^2^ effect sizes.

Next, we performed a specification search. We examined the critical ratios for each of the regression paths and removed the regression path with the smallest critical ratio. We compared the fit of the resulting model to that of the full structural regression model to ensure there had not been a statistically significant change in fit. We continued this process for subsequent regression paths until just before the fit of the model became statistically significantly worse than the full structural model. The paths removed (in order) were: Personal Characteristic/Condition Loss => Climate Change Risk Perception, Personal Characteristic/Condition Loss => Coping, Posttraumatic Symptoms => Posttraumatic Growth, Posttraumatic Symptoms => Climate Change Risk Perception, and Coping => Climate Change Risk Perception. Figure 3 presents the final model, Model 4. Each of the paths was statistically significant (*p* < 0.001). Table 4 shows that all fit indices were acceptable. The fit of the final model and full structural regression model were not statistically significantly different.

To confirm the fit of the final structural model, Model 4, we used the data from the second sample of 343 individuals. The fit indices, shown in Table 4, indicate an excellent overall fit. Figure 2 shows the values for the path coefficients and the R^2^ for each endogenous variable. All structural paths were statistically significant (*p* < 0.001).

**Figure 4:**
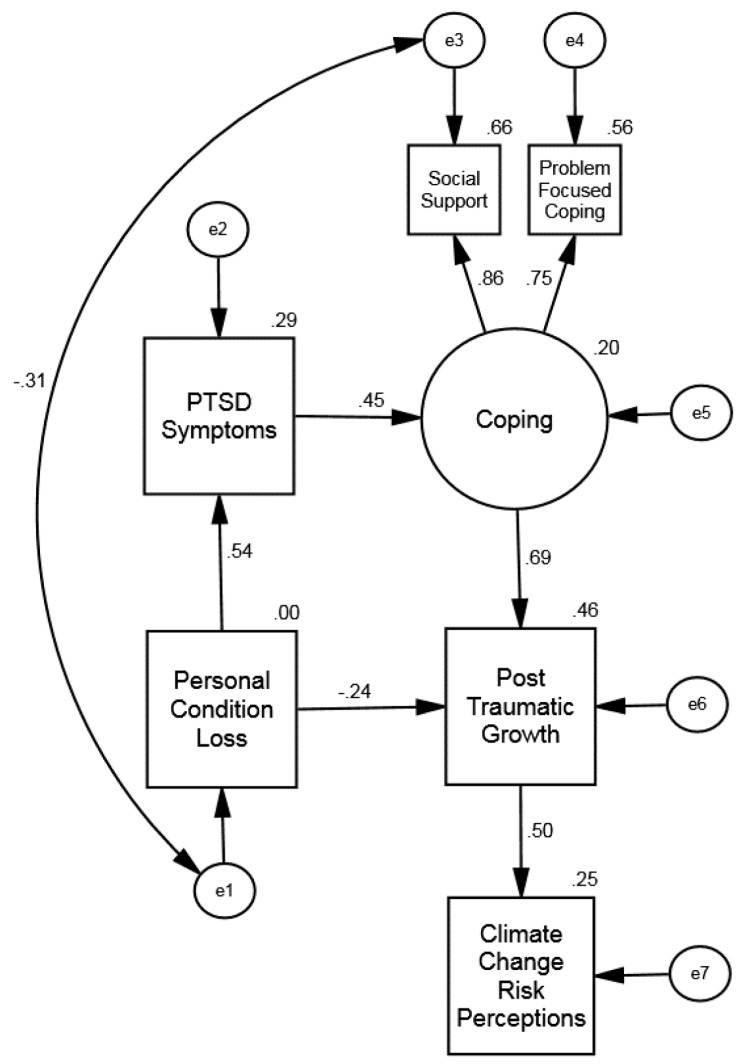
**(Study 2, Fiji)**. Confirmatory Sample Structural Equation Model: Influence of Resource Loss on Posttraumatic Symptoms, Coping, Posttraumatic Growth, and Climate Change Risk Perception. The offset values on endogenous variables are R^2^ effect sizes.

The final model shows that resource loss was positively associated with posttraumatic stress symptoms and posttraumatic growth (see Figure 4). The more loss an individual experienced, the greater their potential for both posttraumatic symptoms and posttraumatic growth. The experience of posttraumatic symptoms was, in turn, associated with higher levels of coping. More coping was associated with greater posttraumatic growth. Resource loss was also directly associated with opportunities for posttraumatic growth, outside of the effect mediated by posttraumatic symptoms and coping. Finally, posttraumatic growth has a positive direct relationship with climate change risk perception, with higher levels of posttraumatic growth relating to a higher degree of perceived risk due to climate change.

## 7. General Discussion

This two-study paper developed a model of climate change risk perception with survivors of Super Typhoon Haiyan in the Philippines and confirmed the model with survivors of Cyclone Winston in Fiji. The final model shows that resource loss was directly associated with posttraumatic stress symptoms, which mediated the relationship of resource loss with coping. Resource loss was also negatively related to social support. This finding suggests that, while resource loss could have a negative impact on coping resources, such as the accessibility of social support, insofar as resource loss is associated with posttraumatic stress, it could also activate problem-focused and relationship-focused coping behaviors [20,26]: the more problem-focused coping that occurred, the greater the posttraumatic growth. Resource loss also created opportunities for posttraumatic growth outside of the effect mediated by problem-focused coping. Finally, posttraumatic growth had a positive direct effect on climate change risk perception, with higher levels of posttraumatic growth being associated with higher levels of climate change risk perception.

Why did posttraumatic growth play a predominant role in climate change risk perception? Posttraumatic growth involves reflection and clarification of values and life priorities [37,38,39]. Because traumatic events like those studied in this paper can create extensive damage and extreme hardship for individuals and communities, survivors may experience cognitive disequilibrium, which involves positive reinterpretation and accommodation of the stressor. Cognitive disequilibrium may serve as “a salutatory if sometimes discomforting factor that motivates people to reexamine their understanding of a situation or circumstance, embrace broader and alternative views, and emerge with a fuller understanding and appreciation of the matter at issue” [40] (p. 420). The experience of life threat, losing property and other essential resources necessary for survival, and facing and enduring a lengthy recovery may result in a new understanding of risks and heightened concern associated with climate change. Research is needed to test this possibility fully. Because experiencing success in taking action to reduce stress and managing negative emotions via problem-focused coping may help promote posttraumatic growth, future research should examine how changes in posttraumatic growth over time may relate to climate change risk perception and behavioral adaptation [10,37,38,39].

These findings support and extend research concerning processes that influence risk perception, and have implications for post-disaster recovery programs and programs promoting preparedness. The protective action decision model states that situational (e.g., physical cues, risk communication), personal characteristic (e.g., age, education, knowledge, affect, personal experience), and social contextual variables (e.g., family size, involvement in community) influence risk perception and taking protective action [41]. The experiences during these storms were tangible and vivid and likely contributed to a new understanding. Affect, knowledge, and experience also play central roles in appraising risk events. Our findings show how physical cues (e.g., damage or destruction of homes, businesses, and infrastructure) and psychological variables (e.g., stress, coping, and posttraumatic growth in response to the disaster) are associated with climate change risk perception [10,42,43]. The findings support research showing that affective responses may serve as a motivating factor such that people are more likely to take protective action to mitigate damage and threat exposure when they perceive a threat is likely and if mitigation efforts will be effective and practical [44,45].

Disaster threats associated with climate change present an existential crisis for nations that may have to relocate completely and experience corresponding cultural changes when relocating from their homeland. For example, sea level rise threatens the existence of low-lying island nations, such as Tuvalu, Kiribati, Maldives, portions of Tonga, and coastal areas of mainland communities [46]. Importantly, research is needed to consider how climate change risk perception may influence subsequent post-disaster intervention and recovery efforts, as well as local and international efforts to address climate change. Research should also examine how might climate change risk perception may motivate survivors, politicians, and disaster relief agencies to take new proactive measures to mitigate the effects of climate change. Research is needed to critically examine the impact of the climate change threat on mental health in affected locations and to develop effective mental health and public health interventions [47]. Finally, research is needed to explore how the experiences of those directly impacted and who face an existential threat may inform those who perceive climate change as distant or question its validity [11,48].

There are several limitations to the present studies. These findings may not generalize to all persons affected by Super Typhoon Haiyan or Cyclone Winston because a purely random sampling strategy was not employed across all areas. We do not know if prior mental health issues or social desirability may have influenced responses. The studies were conducted four to five weeks after the storms made landfall, and research is needed to explore how climate change risk perception may evolve through the passage of time. For example, prior research shows that the influence of personal experience on disaster preparedness and risk perception may dissipate over time [24]. People with higher levels of emotional distress as a result of a prior hurricane were better prepared for a new hurricane threat four years later, but emotional distress did not influence preparation seven years later. Emotional distress may be less likely to influence risk perception over time because symptoms may diminish, information may be less cognitively accessible, and they may be less likely to be activated by relevant environmental cues [24]. Research is needed to extend these findings with a sample of participants who did not directly experience a disaster threat whose magnitude was associated with climate change. Future work examining this model could also explore aspects of risk perception not directly measured in this study.

## 8. Conclusions

A critical step for climate change mitigation is acknowledging the threat to public health and life on Earth. Our findings highlight the importance of key variables for climate change risk perception, and especially the central role of posttraumatic growth, which involves reflecting on that which gives life meaning and value. Importantly, the generalizability of the model is enhanced, given that the model developed in the Philippines was confirmed in Fiji.

There are several practical implications of these findings. First, efforts to educate the public about climate change should present information in a manner that vividly conveys how the threat can adversely impact their community. For example, information could vividly show how climate change can create a loss of resources (e.g., home, income, employment, social support), psychological distress, and damage to community infrastructure. This approach can help people better understand how climate change may directly impact their lives. Disaster response organizations may also use this approach to promote preparedness for disaster threats. Second, educational efforts could encourage the public to reflect on that which gives life meaning and value. This approach may help people consider and better understand the existential threat the climate crisis can pose. Third, educational efforts might encourage people who have direct experience with climate change to detail how climate change is adversely impacting their life and what motivated them to take action. For example, the first author with colleagues are developing an instructional program to teach youth in South Pacific Island nations how to use video and photography to create a “my climate change story.” With compelling imagery and narrative, these stories not only document the impacts of climate change but can also educate and inspire others to take action and promote empathy. Research is needed to examine the effectiveness of these approaches.

## Figures and Tables

**Table 1:** **(Study 1: Philippines)**. Means, Standard Deviations, and Correlations between Measured Variables for Initial Sample (*N* = 160) and Confirmatory Sample (*N* = 161).

Variable	1	2	3	4	5	6	7	8
Mean	1.88	2.14	48.73	1.95	3.71	3.60	4.70	4.99
Standard Deviation	1.77	0.87	17.86	1.14	0.71	0.66	0.98	1.17
1. Personal Condition loss		0.65	0.47	0.38	−0.12	0.07	0.25	0.22
2. Object/Energy Loss	0.54		0.56	0.46	−0.08	0.04	0.23	0.28
3. Posttraumatic Stress	0.39	0.45		0.69	0.21	0.25	0.34	0.26
4. Somatic Problems	0.31	0.38	0.71		0.14	0.14	0.23	0.19
5. Social Support	−0.27	−0.04	0.25	0.19		0.57	0.35	0.20
6. Problem-Focused Coping	0.02	0.02	0.27	0.13	0.58		0.56	0.31
7. Posttraumatic Growth	0.21	0.36	0.36	0.26	0.33	0.38		0.57
8. Climate Change Risk Perception	0.08	0.15	0.19	0.06	0.07	0.24	0.46	
Mean	1.69	1.98	48.04	1.94	3.77	3.66	4.70	4.98
Standard Deviation	1.11	0.85	16.77	1.13	0.71	0.61	1.03	1.09

*Note: The initial sample is below the diagonal. The confirmatory sample is above the diagonal.*

**Table 2:** **(Study 1: Philippines).** Fit Indices for Measurement and Structural Regression Models.

Sample	Model	X^2^	*df*	*p*	RMSEA	SRMR	CFI
#1, *N* = 160	1 (Measurement Model)	36.342	12	<0.001	0.113	0.059	0.940
	2 (E2 ⇔ E6)	15.197	11	0.174	0.049	0.032	0.990
	Difference	21.145	1	<0.001			
	3 (Full Structural Regression)	15.197	11	0.174	0.049	0.032	0.990
	4 (Final Structural Model)	22.124	16	0.139	0.049	0.050	0.985
	Difference	6.927	5	0.226			
#2, *N* = 161	4 (Final Structural Model)	18.466	16	0.297	0.031	0.051	0.995

*Note: Sample #1 was used for initial analyses and specification searches. Sample #2 was used solely for confirmatory purposes*.

**Table 3:** **(Study 2: Fiji)**. Means, Standard Deviations, and Correlations between Measured Variables for Initial Sample (*N* = 343) and Confirmatory Sample (*N* = 343).

Variable	1	2	3	4	5	6	7	8
Mean	1.95	2.97	51.33	1.44	3.46	3.80	5.70	5.66
Standard Deviation	1.19	0.88	20.14	1.18	0.75	0.83	1.10	1.16
1. Personal Condition loss		0.44	0.54	0.43	−0.01	0.12	−0.10	−0.04
2. Object/Energy Loss	0.46		0.46	0.20	0.18	0.14	0.15	0.10
3. Posttraumatic Stress	0.64	0.50		0.50	0.27	0.36	0.21	0.18
4. Somatic Problems	0.45	0.36	0.55		−0.01	0.11	−0.10	−0.05
5. Social Support	0.03	0.18	0.18	−0.04		0.62	0.57	0.32
6. Problem-Focused Coping	0.18	0.24	0.34	0.13	0.61		0.47	0.27
7. Posttraumatic Growth	−0.02	0.17	0.14	−0.19	0.46	0.40		0.50
8. Climate Change Risk Perception	0.03	0.18	0.15	−0.93	0.36	0.29	0.57	
Mean	1.96	2.97	49.90	1.34	3.52	3.83	5.68	5.60
Standard Deviation	1.20	0.83	22.07	1.19	0.71	0.84	1.13	1.18

*Note: The initial sample is below the diagonal. The confirmatory sample is above the diagonal.*

**Table 4:** **(Study 2: Fiji)**. Fit Indices for Measurement and Structural Regression Models.

Sample	Model	X^2^	*df*	*p*	RMSEA	SRMR	CFI
#1, *N* = 343	1 (Measurement Model)	16.549	3	0.001	0.115	0.036	0.978
	2 (E2 ⇔E6)	6.036	2	0.049	0.077	0.014	0.993
	Difference	10.513	1	0.001			
	3 (Full Structural Regression)	6.036	2	0.049	0.077	0.014	0.993
	4 (Final Structural Model)	13.190	7	0.068	0.051	0.035	0.990
	Difference	7.154	5	0.209			
#2, *N* = 343	4 (Final Structural Model)	9.385	7	0.226	0.032	0.030	0.996

*Note: Sample #1 was used for initial analyses and specification searches. Sample #2 was used solely for confirmatory purposes.*

## Data Availability

The data presented in this study are available upon request from the corresponding author.
